# MicroRNA-124-3p inhibits cell migration and invasion in bladder cancer cells by targeting ROCK1

**DOI:** 10.1186/1479-5876-11-276

**Published:** 2013-11-02

**Authors:** Xianglai Xu, Shiqi Li, Yiwei Lin, Hong Chen, Zhenghui Hu, Yeqing Mao, Xin Xu, Jian Wu, Yi Zhu, Xiangyi Zheng, Jindan Luo, Liping Xie

**Affiliations:** 1 Department of Urology, the First Affiliated Hospital, Zhejiang University, Hangzhou, Zhejiang Province, China

**Keywords:** miR-124-3p, ROCK1, Bladder cancer, Migration, Invasion

## Abstract

**Background:**

Increasing evidence has suggested that dysregulation of certain microRNAs (miRNAs) may contribute to human disease including carcinogenesis and tumor metastasis in human. miR-124-3p is down-regulated in various cancers, and modulates proliferation and aggressiveness of cancer cells. However, the roles of miR-124-3p in human bladder cancer are elusive. Thus, this study was conducted to investigate the biological functions and its molecular mechanisms of miR-124-3p in human bladder cancer cell lines, discussing whether it has a potential to be a therapeutic biomarker of bladder cancer.

**Methods:**

Three human bladder cancer cell lines and samples from ten patients with bladder cancer were analyzed for the expression of miR-124-3p by quantitative RT--PCR. Exogenetic overexpression of miR-124-3p was established by transfecting mimics into T24, UM-UC-3 and J82 cells, after that cell proliferation and cell cycle were assessed by MTT assay, flow cytometry and Colony-forming assay. Cell motility and invasion ability were evaluated by wound healing assay and transwell assay. Tissue microarray, and immunohistochemistry with antibodies against ROCK1, MMP2 and MMP9 was performed using the peroxidase and DAB methods. The target gene of miR-124-3p was determined by luciferase assays, quantitative RT--PCR and western blot. The regulation of epithelial-to-mesenchymal transition by miR-124-3p was analyzed by western blot.

**Results:**

miR-124-3p is frequently down-regulated in bladder cancer both in three bladder cancer cell lines, T24, UM-UC-3, J82 and clinical samples. Overexpression of miR-124-3p induced G1-phase arrest in T24, UM-UC-3 and J82 cell lines and suppressed cell growth in colony-forming assay. miR-124-3p significantly repressed the capability of migration and invasion of bladder cancer cells. In addition, ROCK1 was identified as a new target of miR-124-3p. ROCK1, MMP2, MMP9 were up-regulated in bladder cancer tissues. Furthermore, we demonstrated miR-124-3p could inhibit bladder cancer cell epithelial mesenchymal transfer, and regulated the expression of c-Met, MMP2, MMP9.

**Conclusions:**

miR-124-3p can repress the migration and invasion of bladder cancer cells via regulating ROCK1. Our data indicate that miR-124-3p could be a tumor suppressor and may have a potential to be a diagnostics or predictive biomarker in bladder cancer.

## Background

Bladder cancer (BCa) is listed as the 7th of the most common cancer in men and the 17th in women worldwide [1]. It is associated with accumulation of chromosomal anomalies, genetic polymorphisms and epigenetic changes [2]. Approximately 70% of bladder tumors are classified as nonmuscle invasive tumors, whereas the remaining cases have invasive potential. Most patients with nonmuscle invasive tumors are treated by transurethral resection, with a 40% to 80% risk of recurrence and a 10% to 27% chance of progressing to muscle-invasive, regional, or metastatic disease. About 25% of patients with newly diagnosed bladder cancer have muscle invasive disease, the vast majority presents with cancers of high histological grade. Furthermore, almost 50% of bladder cancer with muscle-invasive already have asymptomatic and nonpalpable distant metastases [3]. The limitations of established bladder cancer biomarkers requires us to identify better molecular parameters that could be clinically useful for diagnosis and prognosis, in particular, for the high-risk patient groups that are usually at high risk of progression, recurrence and metastasis.

MicroRNAs (miRNAs) are small (approximately 22-nucleotides), endogenous, noncoding RNAs, functioning as negative regulators of gene expression through antisense complimentarily to their target messenger RNAs. miRNAs could induce post-transcriptional gene depression by the repressing of the translation or promoting the degradation of specific mRNAs [4,5]. Increasing evidence revealed that disordered expression of miRNA contributes to the initiation and progression of human cancer [6]. It has been shown that miRNAs participate in human carcinogenesis as either tumor suppressors or oncogenes. Specifically, aberrant regulation of miRNAs in bladder cancer was suggested to contribute to bladder tumorigenesis [7,8]. Moreover, the profile of altered miRNAs appears distinct between noninvasive (including carcinoma in situ [CIS]) and muscle-invasive high-grade cancers [9]. Some of those miRNAs could be potential biomarkers for bladder cancer in diagnosis, prognosis predicting and treatment target. For instance, miR-143, miR-222, and miR-452 detected in urinary specimens were clinically useful for noninvasive bladder cancer diagnostics [10], and miR-9, miR-182, and miR-200b was found to be related to bladder tumor aggressiveness and survival [11].

The miR-124-3p was detected in 46 animal species from Caenorhabditis to Homo sapiens [12] and it is inevitable in neurogenesis [13]. Recent reports further demonstrated that decreased expression of miR-124-3p is related to carcinogenesis. The epigenetic silencing of miR-124-3p suggests its potential tumor suppressive function in glioma, oral squamous cell carcinomas, hepatocellular carcinoma (HCC) and breast cancer [14-17]. miR-124-3p regulates cell cycle and motility by targeting to CDK6 and ITGB1 [15,18]. A study reported that miR-124-3p was deregulated in bladder cancer tissues and cell lines because of methylation, by which they believed that it could serve as a diagnostic biomarker for BCa detection. Restoration of miR-124 may be an effective anticancer therapy [19]. However, the specific function of miR-124-3p in bladder cancer progression, especially its molecular mechanisms by which miR-124-3p exerts its functions and modulates the malignant phenotypes of bladder cancer cells, has not been fully understood.

In this study, we demonstrated the pathologically down-regulation of miR-124-3p in both bladder cancer specimens and cell lines. Ectopic overexpression of miR-124-3p not only repressed cell motility and invasion capability, but also triggered G1-phase arrest of human bladder cancer cells. More importantly, we illustrated that miR-124 directly target the Rho-associated, coiled-coil containing protein kinase 1 (ROCK1). In addition, we provide evidence that miR-124-3p appears to play an important role in epithelial mesenchymal transfer (EMT), and modulates the expression of genes which promote cancer metastasis, such as MMP2, MMP9, c-Met. Thus, our findings provide valuable clues toward understanding the specific tumor suppressive function and the regulatory mechanisms of miR-124-3p in human bladder cancer. Further investigation may present miR-124-3p as an effective therapeutic biomarker in the future.

## Materials and methods

### Oligonucleotide transfection

miR-124-3p mimic (named as miR-124-3p), miR-124-3p inhibitor, negative control duplex (named as NC) and siRNA against ROCK1 (named as siROCK1), were all synthesized by GenePharma (Shanghai, China), were applied for transfection. Oligonucleotide transfection was performed using Lipofectamine 2000 reagents (Invitrogen, Carlsbad, CA, USA) according to the manufacturer’s protocol. The sequences were listed in Table 1.

**Table 1:** Oligonucleotide sequences

**Name**^**a**^	**Sequence (5′ to 3′)**^**b**^
miR-124-3p mimics (sense)	UAAGGCACGCGGUGAAUGCC
NC (sense)	ACUACUGAGUGACAGUAGA
siROCK1 (sense)	GAAGAAACAUUCCCUAUUCTT
U6-F	TGCGGGTGCTCGCTTCGGCAGC
ROCK1-F	AACATGCTGCTGGATAAATCTGG
ROCK1-R	TGTATCACATCGTACCATGCCT
miR-124-3p-F	TAAGGCACGCGGTGAATGCC
GAPDH-F	ACAACTTTGGTATCGTGGAAGG
GAPDH-R	GCCATCACGCCACAGTTTC
ROCK1-utr-F	CGTTGCATTGTCCTTTTA*GTGCCTTA*ATTTGAGATAATTATTTTACG
ROCK1-utr-R	TCGACGTAAAATAATTATCTCAAATTAAGGCACTAAAAGGACAATGCAACGAGCT
ROCK1-mut-F	CGTTGCATTGTCCTTTTACACGGAATATTTGAGATAATTATTTTACG
ROCK1-mut-R	TCGACGTAAAATAATTATCTCAAATATTCCGTGTAAAAGGACAATGCAACGAGCT

^a^F, forward primer; R, reverse primer.

^b^Target sites are in italic type; Mutated target sites are underlined.

### Cell lines and cell culture

The human non-malignant cell line SV-HUC1, human bladder cancer lines T24, UM-UC-3, J82 cells and HEK293T cells were purchased from Shanghai Institute of Cell Biology, Chinese Academy of Sciences and were cultured in RPMI 1640 medium supplemented with 10% fetal bovine serum under an humidified air atmosphere of 5% CO_2_ at 37°C.

### Human clinical samples

Thirteen paired bladder cancer tissues and adjacent non-tumorous bladder mucosal tissues were obtained from patients undergoing radical cystectomy at the First Affiliated Hospital of Zhejiang University, China, from January to June in 2011 and September to October in 2013. All the patients signed informed consent and the study was approved by the Ethical Committee of First Affiliated Hospital of School of Medicine of Zhejiang University. Tissue samples were trimmed and snap frozen in liquid nitrogen until use. Tissue samples were immediately frozen in liquid nitrogen until RNA extraction.

### RNA isolation and quantitative real-time PCR

Total RNA from tissue samples and cultured cells was isolated using TRIzol reagent (Takara, Dalian, China). Before performing Quantitative RT-PCR assays (qPCR), RNA was reverse transcribed into miRNA cDNA and total cDNA using One Step PrimeScript miRNA cDNA Synthesis Kit (Takara, Dalian, China) and PrimeScript RT reagent Kit (Takara, Dalian, China), respectively. The mRNA and miRNA expression levels were detected by qPCR with Applied Biosystems 7500 Fast Real-Time PCR System real-time PCR System (Applied Biosystems, Carlsbad, USA) with SYBR Premix Ex Taq (Takara, Dalian, China) according to the manufacturer’s instructions and were normalized versus GAPDH mRNA and small nuclear RNA U6, respectively. The Ct value of miR-124-3p and ROCK1 was quantified with the 2-∆∆Ct method.

### Dual-luciferase reporter assay

Bioinformatic analysis through the online software programs TargetScan (http://www.targetscan.org/) found that ROCK1, a potential metastasis promoter, is probably a direct target of miR-124-3p. Two oligonucleotide pairs that contained the desired miRNA target region and mutant miRNA target region were designed and ordered from Sangon, Shanghai, China. Two oligonucleotide pairs were annealed at 90°C for 3 minutes, then transfer to 37°C for 15 minutes. The couple of annealed oligonucleotides were then ligated into pmirGLO, Dual-Luciferase miRNA Target Expression Vector (Promega, USA), between the SacI and SalI sites. Both insertions were sequenced to prevent any mutant. HEK293T cells were plated in 24-well plates and co-transfected with 50 nM miR-124-3p or NC RNA and with 100 ng of the pmirGLO. The relative Luciferase activity was measured using the Dual-Luciferase Reporter Assay System (Promega, USA) 48 h after co-transfection.

### Cell cycle analysis by flow cytometry

Cells were harvested 48 h after transfection, washed with PBS and fixed in 75% ethanol at −20°C. After overnight fixation, cells were washed with PBS and stained with DNA Prep Stain (Beckman Coulter, Fullerton, CA) for 30 min. Cell cycle analysis was performed by BD LSRII Flow Cytometry System with FACSD via software (BD Bioscience, Franklin Lakes, USA). Data was analyzed with ModFit LT software package.

### MTT assay

Approximately 3 × 10^3^ T24, UM-UC-3 or J82 cells were plated in each well of a 96-well plate. After an overnight incubation, the cells were transfected with the NC, miR-124-3p or siROCK1 for 24–96 h. The RNA concentration ranged from 25 to 75 nM. At various times following treatment, the medium was removed and MTT (20 μl of 5 mg/mL, Sigma–Aldrich, St. Louis, USA) was added to each well. The 96-well plates were incubated at 37°C for 4 h. The plates were centrifuged, and the formazan precipitates were dissolved in 150 μl of dimethyl sulfoxide. The absorbance of the solution was measured at 490 nm using a MRX II absorbance reader (DYNEX Technologies, Chantilly, Virginia, USA).

### Colony-forming assay

The cells were harvested 24 h after RNA treatment (50 nM of NC or 50 nM of miR-124-3p). Then the cells were resuspended in RPMI 1640 medium supplemented with 10% FBS and plated at a density of 500 cells/well in 6-well plate. The cultures were maintained under standard culture conditions for 14 days. The estimation of colonies was performed after the colonies were fixed with absolute methanol for 15 min and stained with crystal violet for 20 min.

### Migration and invasion assays

Cell migration and invasion were assayed using a transwell chamber (Millipore, USA) with and without Matrigel (BD, Franklin Lakes, USA). For the invasion assay, a transwell chamber was placed into a 24-well plate and was coated with 30 μl Matrigel and was incubated for forty minutes at 37°C. In both transwell assay, cells, 48 hours after transfected, were trypsinized and seeded in chambers at the density of 8 × 10^4^ cells per well and cultured in medium with RPMI 1640 medium with 2% serum, while 600 μl of 10% FBS–1640 was added to the lower chamber. Twenty-four hours later, migrated cells were fixed with 100% methanol for 30 min. Non-migrated Cells were removed by cotton swabs. Then cells on bottom surface of the membrane were stained by crystal violet for 20 min. Cell images were obtained under a phase-contrast microscope (Olympus, Tokyo, Japan).

### Wound healing assays

Cells were grown to basically 100% confluence in 6-well plates after RNAs transfection. The cell monolayers were wounded by scraping them with a micropipette tip. The spread of wound closure was observed after 24 h. Photographs were taken under a phase-contrast microscope (Olympus, Japan) immediately following or 24 h after wounding. These experiments were repeated three times.

### Western blotting analysis

Cells were harvested at 48 h following various treatments as described above, lysed and quantified by a BCA kit (Thermo, USA). Proteins were separated by 10% SDS–PAGE and transferred to polyvinylidene difluoride (PVDF) membranes. The membranes were then blocked with 5% non-fat milk and incubated overnight with antibodies against ROCK1, c-Met, MMP2, MMP9, fibronectin, vimentin, N-cadherin and β-catenin (Epitomics, Burlingame, USA), GAPDH (Sangon Biotech, Shanghai, China) at dilutions specified by the manufacturer’s protocol. After washed in TBS for three times, the membranes were incubated for 1 h with horseradish peroxidase-conjugated goat anti-rabbit secondary antibody at the 1:5000 dilution. After another three times of washing in TBS, the bound secondary antibody was detected using an enhanced chemiluminescence (ECL) system (Pierce, Biotechnology Inc., Rockford, USA).

### Immunohistochemical (IHC) staining

Tissues microarray, which contains small representative tissue samples from 31 of different cases and their paired non-tumor tissues (NT), was deparaffinized, rehydrated, and microwave-heated in sodium citrate buffer (10 mM, pH 6.0) for antigen retrieval. Bovine Serum Albumin was used for blocking. The slides were incubated with ROCK1 or MMP9 antibody (Epitomics, Burlingame, USA) or MMP2 (Santa Cruz), respectively, overnight at 4°C at the optimal dilution, and incubated with a HRP-conjugated secondary antibody at room temperature for 1 h. DAB was applied for color development, and dark brown was considered positive staining. The strength of positivity was semi-quantified by comprehensively considering staining intensity and the proportion of positive cells.

### Statistical analysis

The data were expressed as mean ± SD of three independent experiments. Student’s t-test was used to compare test groups with negative control ones, while Two-way ANOVA was used to contrast the differences among three or more experimental groups. Statistical analysis was performed using GraphPad Prism version 5 for Windows and P < 0.05 was considered to be statistically significant.

## Result

### miR-124-3p is frequently down-regulated in bladder cancer both in BCa clinical samples and cell lines

**Figure 1:**
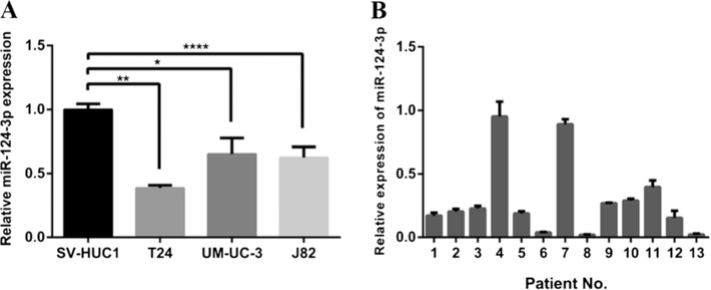
**Expression patterns of miR-124-3p in urinary bladder cancer tissues and BCa cell lines. (A)** qPCR detection of miR-124-3p in T24, UM-UC-3 and J82 cell lines versus SV-HUC-1. It was normalized with U6 snRNA in quantitative real-time RT-PCR analysis. **(B)** The miR-124-3p levels were determined in 10 surgical specimens of human bladder cancer tissues and were normalized to that in the adjacent normal bladder tissues, which is defined as 1. *P <0.05, **P < 0.01, ****P < 0.0001.

To investigate the role of miR-124-3p in human bladder cancer, we first compared the expression levels between three bladder cancer cell lines (T24, UM-UC-3, J82) and non-malignant cell line SV-HUC1 by real-time RT-PCR. The result showed that the expression levels of miR-124-3p in all three cell lines were significantly reduced at different degrees compared with SV-HUC1 (Figure 1A).

We further compared the expression levels of miR-124-3p between bladder carcinomas tissue samples and paired adjacent normal mucosal tissues from 13 cases of bladder cancer patients. We found that miR-124-3p expression levels were decreased in cancerous tissues compared to their corresponding non-cancerous controls (P < 0.001), with 11 out of 13 exhibiting over 50% reduction (Figure 1B). It was consistent with data from a previous study [19]. From these results, we speculated that miR-124-3p may play some important roles in human bladder cancer.

### miR-124-3p induces G1-phase arrest and inhibits clonogenicity in bladder cancer cell lines

**Figure 2:**
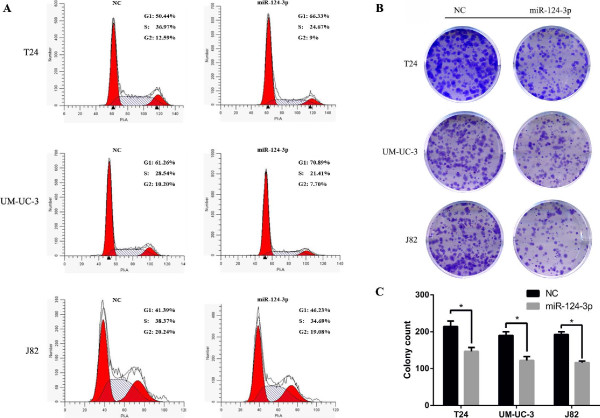
**miR-124-3p induces G1-phase arrest and inhibits clonogenicity in bladder cancer cell lines. (A)** The T24, UM-UC-3 and J82 cells, transfected with NC RNA or miR-124-3p, were subjected to flow cytometry for cell cycle analysis. Three independent experiments were performed in each group. **(B)** Decreased clonogenicity in bladder cancer cells treated with miR-124-3p (Representative wells were presented). **(C)** The colony count was significantly lower for miR-124-3p treated group compared with NC treated group (P < 0.05).

First, we performed MTT Assay to investigate whether miR-124-3p has a biological function in proliferation of cancer cells. No significant difference was observed between NC group and miR-124-3p treated group in MTT assay (Data not shown). This experiment was repeated three times. We performed flow cytometry to confirm that exogenetic overexpression of miR-124-3p do not suppresses bladder cell-line growth. To our surprise, a significant accumulation of cells in the G1 phase was observed in three cell lines after miR-124-3p treatment (p < 0.05). This increase in G0/G1 cell population was accompanied with a concomitant decrease of cell number in S phase and G2–M phase in three bladder cancer cell lines (Figure 2A). To further identify whether miR-124-3p affects cell growth, colony-forming assay was used to evaluate the effects of miR-124-3p on cell proliferation. Notably, three cell lines showed a dramatic inhibition of clonogenicity in the miR-124-3p-treated group (p < 0.05; Figure 2B, C). The above results suggest that miR-124-3p regulates proliferation in bladder cancer cells.

### Cell migration and invasion is inhibited by miR-124-3p in human bladder cancer

**Figure 3:**
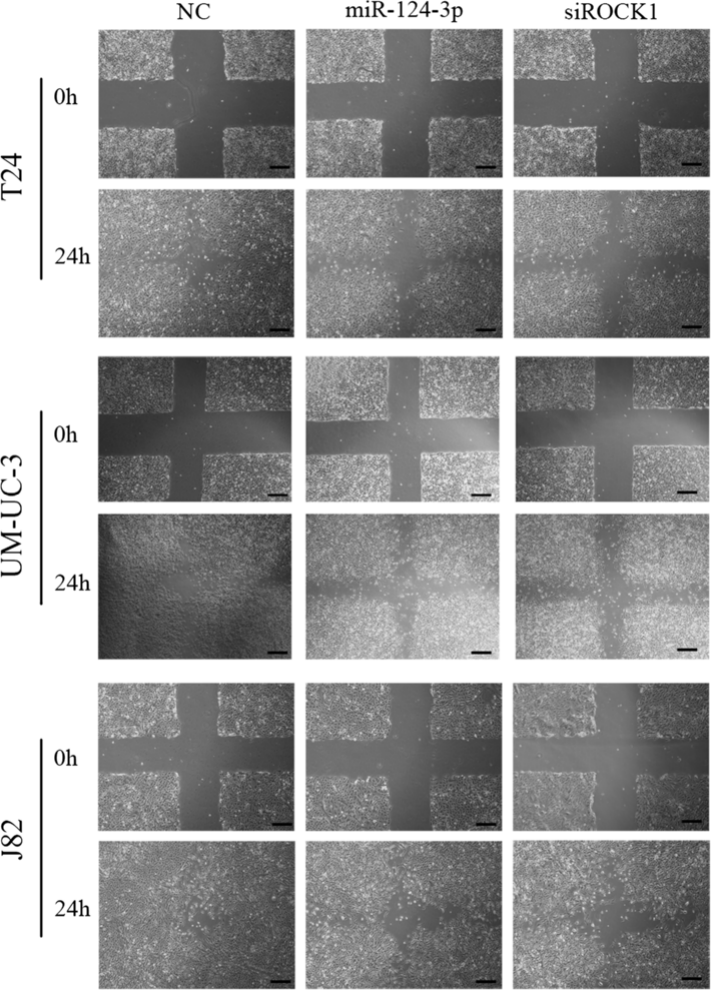
**Forced expression of miR-124-3p suppresses cell motility in wound healing assay.** T24, UM-UC-3 and J82 cells were transfected with NC, miR-124-3p mimics or siROCK1 and were performed wound healing assays with a 24-h recovery period; Scale bars = 500 μm.

**Figure 4:**
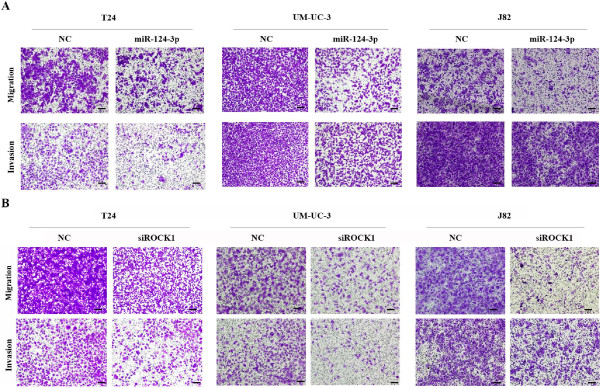
**Forced expression of miR-124-3p and silenced expression of ROCK1 suppresses cell migration and invasion in transwell assay. (A)** T24, UM-UC-3 and J82 cells were transfected with NC or miR-124-3p mimics. Cell migration and invasion were assessed after 24 hours incubation by transwell assay. **(B)** T24, UM-UC-3 and J82 cells were transfected with NC or siROCK1. Cell migration and invasion were evaluated after 24 hours incubation by transwell assay; Scale bars = 100 μm.

Considering T24, UM-UC-3 and J82 cells are highly metastatic cell, we wonder whether decreased miR-124-3p has any effect on migration and invasion capacity. T24, UM-UC-3 and J82 cells were therefore transfected with miR-124-3p mimics and incubated 48 h before the wound healing assay was taken. Forced expression of miR-124-3p in all three cells led to retarded wound closing compared to NC groups (Figure 3). Migration chamber assay was used to verify the biological function of miR-124-3p in bladder cancer cell migration. After 24 h, the migrating cells were fixed, stained and observed microscopically. As the representative micrographs clearly demonstrate, miR-124-3p overexpression led to potent inhibition of cell migration (Figure 4A).

Likewise, invasion capability of bladder cancer cells transfected with NC or miR-124-3p was evaluated by Matrigel invasion chamber assay. As our expected, transfected miR-124-3p mimics in T24, UM-UC-3 and J82 can notably repress their invasion ability compared with NC groups (Figure 4A).

### miR-124-3p directly targets ROCK1 3′-UTR

**Figure 5:**
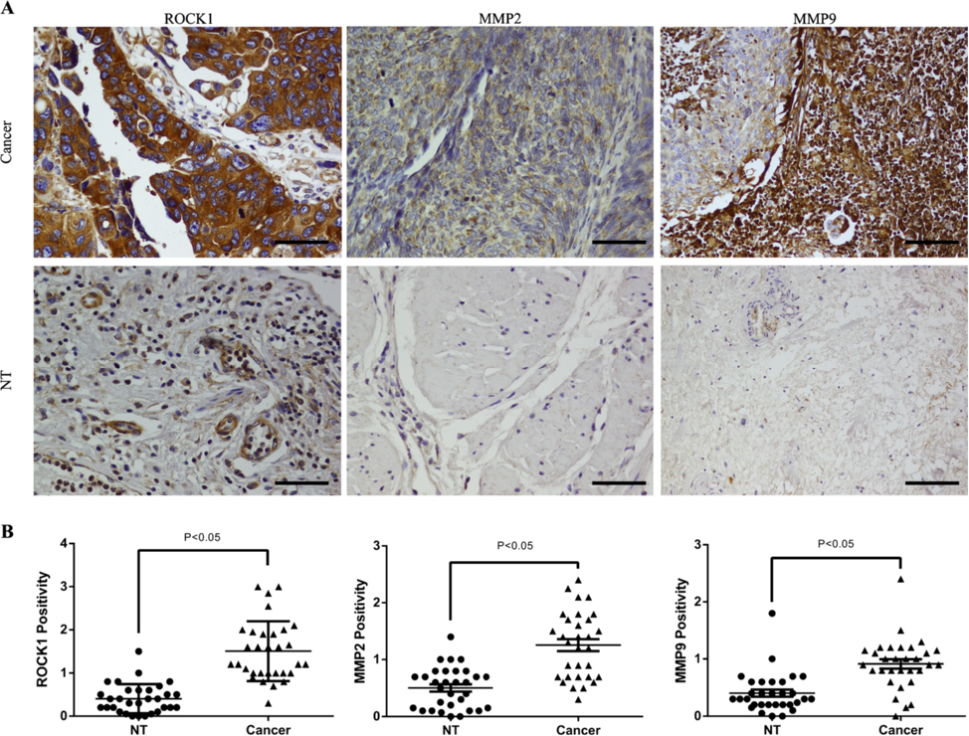
**IHC analysis of ROCK1, MMP2 and MMP9 expression pattern in bladder cancer tissues. (A)** Representative images of IHC staining for ROCK1, MMP2 and MMP9 in human bladder cancer tissues and NT tissues were captured at 400× magnification. Scale bars = 100 μm. **(B)** Positive strength of ROCK1, MMP2 and MMP9 were significantly higher in bladder cancer tissues compared with paired non-tumor tissues; P < 0.05.

It is generally understood that miRNAs execute post-transcriptional regulation by binding to the 3′-UTR of their downstream genes. To find the target which is involved in the regulation of cell motility and invasion capability triggered by miR-124, we used bioinformatics prediction software Targetscan (http://http://www.targetscan.org/). Among these thousands of candidates, we focused on ROCK1. It has been demonstrated that the Rho/ROCK pathway participates in regulating cytoskeletal signalling and is crucial for cell motility [20,21]. Firstly, to investigate whether ROCK1 is upregulated in bladder cancer, we detected its expression level in bladder cancer tissues. In our current study, we revealed that ROCK1 was commonly over-expressed in bladder cancer tissues by immunohistochemical staining, comparing with the paired non-tumor tissues (NT) (Figure 5A, B). In order to discover whether miR-124 regulates cell motility and invasion capability via ROCK1 3′-UTR, we performed luciferase reporter assay.

**Figure 6:**
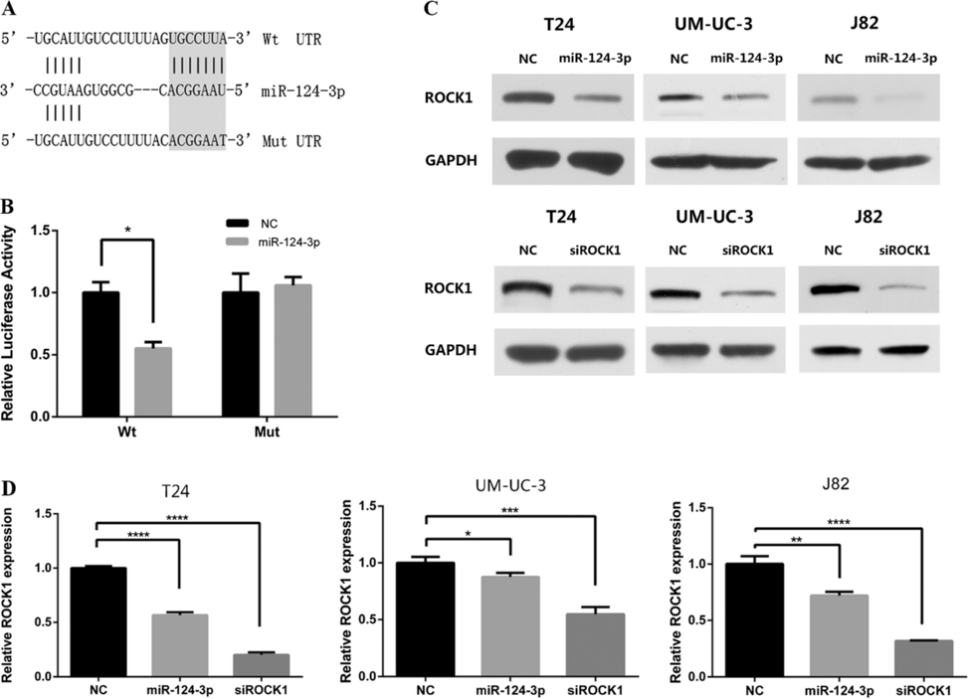
**miR-124-3p directly targets ROCK1. (A)** Oligonucleotides containing the predicted miR-124-3p binding sites in the 3′UTR of ROCK1 mRNA was synthetized, while mutations on the “seed” sequences are designed as below. Alignment between the predicted miR-34a target sites and miR-124-3p is marked with black color. **(B)** HEK293T cells were co-transfected with 50 nM of either miR-124-3p mimics or NC and 100 ng pmirGLO Dual-Luciferase miRNA Target Expression Vector with Wt or Mut 3′-UTR of ROCK1. The relative luciferase activity was measured 48 h after transfection. *P < 0.05. **(C)** The down-expression of ROCK1 was confirmed by western blotting, after miR-124-3p or siROCK1 transfected, GAPDH was used as control. **(D)** Quantitative real-time PCR analysis indicated that the relative mRNA level of ROCK1 was significantly decreased after miR-124-3p or RNAi treatment. GAPDH was used as control. *P < 0.05, **P < 0.01, ***P < 0.001, ****P < 0.0001.

Annealed oligos containing the wild type (WT) of 3′-UTR of ROCK1 or mutated (mut) target site was cloned into pmirGLO Dual-Luciferase Vector (Figure 6A). HEK 293 T cells were transiently transfected with these constructs and miR-124-3p mimics or NC. miR-124-3p mimics significantly suppressed the luciferase activity of reporter genes which contains wild type of 3′-UTR of ROCK1 (Figure 6B). In contrast, the inhibition was fully rescued when all target sites were mutated (Figure 6B).

To further prove if miR-124-3p represses ROCK1 expression in the human bladder cancer intracellular environment, we analysed the changes of ROCK1 expression in T24, UM-UC-3 and J82 after miR-124-3p overexpression. By real-time RT-PCR, we found that the mRNA levels of ROCK1 were notably reduced in miR-124-3p treated groups, compared with that in NC groups (Figure 6D). Meanwhile, western blot assays were taken to evaluate the protein level of ROCK1. The results showed that ROCK1 was also dramatically decreased in protein level after ectopic overexpression of miR-124-3p (Figure 6C).

Collectively, these data supported our speculation that ROCK1 is a direct target of miR-124-3p.

### ROCK1 is involved in miR-124-3p-induced repression of BCa cell migration and invasion

To explore if ROCK1 has similar function as miR-124-3p in bladder cancer cells, RNA approach was used. The silencing of ROCK1 was confirmed by real time RT-PCR and western blot (Figure 6C, D). With the efficient knockdown of ROCK1, we performed MTT assay, flow cytometry and colony-forming assay in T24, UM-UC-3 and J82 cell lines. There was no significant difference in both experiments between siROCK1 groups and NC groups.

Then wound healing assays and transwell assays were taken to observe the function of ROCK1 in bladder cancer cell migration and invasion. T24, UM-UC-3, J82 cells were treated with 50 nM siROCK1, and the siROCK1 groups presented retarded wound closing and less stained cells in wound healing assays and transwell assays, respectively, compared with NC groups (Figure 3 and Figure 4B). The results demonstrated that the silencing of ROCK1 caused significant suppression of the migratory and invasive capability in much the same pattern as miR-124-3p overexpression.

**Figure 7:**
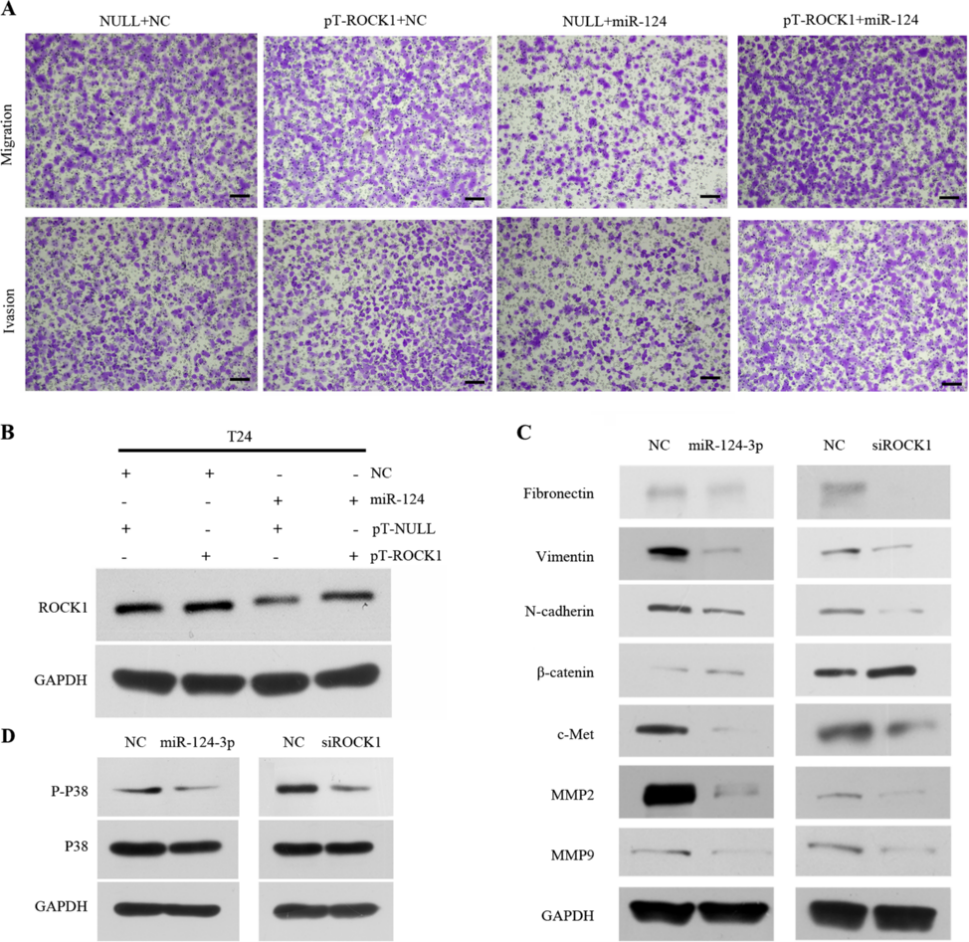
**Forced expression of ROCK1 rescues miR-124-3p-associated decrease in cell migration and invasion. miR-124-3p inhibits T24 EMT. (A)** T24 cells were transfected as in **(B)** and then used in a transwell migration assay (top) or an invasion assay (bottom). Scale bars = 100 μm **(B)** Either miR-124-3p mimics or NC oligos were co-transfected with the pT-ROCK1 or the empty pTarget vector, pT-NULL, into T24 cells. Western blot was then performed to detect the expression of ROCK1. GAPDH was used as control. **(C)** Expression of epithelial markers and mesenchymal markers were compared by western blot between NC and miR-124-3p or siROCK1 groups. GAPDH was used as a loading control. **(D)** Expression of p-p38MAPK and p38MAPK were determined by Western blot analysis.

Next, to investigate if miR-124-3p exerts its function via ROCK1 on migration and invasion of human bladder cancer cells, we ectopically expressed ROCK1 together with miR-124-3p in T24 cells. We inserted the human ROCK1 coding sequence into the pTarget vector without 3′- UTR. Western blot was used to demonstrate that ROCK1 expression was restored after transfection of cells with this pT-ROCK1 construct (Figure 7B). 48 hours after transfected, T24 cells were collected to perform transwell assays. More cells in pT-ROCK1 + miR-124-3p group than NULL + miR-124-3p group showed that overexpression of ROCK1 abrogated the reduction of migration and invasion ability caused by ectopic expression of miR-124-3p in T24 cells (Figure 7A). Altogether, the above results suggest that miR-124-3p reuglates migration and invasion capability of human bladder cancer cells via ROCK1.

### Exogenous overexpression of miR-124-3p inhibits BCa cell EMT

Next, we tried to find the mechanism by which miR-124-3p suppresses cell motility and invasion ability. Since EMT is well known to be involved in invasion and metastasis of cancer cells, we asked whether miR-124-3p can reverse the EMT progression. We evaluated the epithelial and mesenchymal markers by western blot in Negative control group and miR-124-overexpressing T24 cells. After miR-124 was exogenous overexpressed in T24 cells, the expression levels of epithelial marker β-catenin increased (Figure 7C) while the levels of three mesenchymal markers (ie, fibronectin, N-cadherin and vimentin) decreased, (Figure 7C). We also tried to evaluate the expression level of E-cadherin, another epithelial marker. However, E-cadherin could not be detected by western blot in T24 cells. Other molecule promoting cancer cells migration and invasion like c-Met, MMP2, MMP9 were assessed by western blot. As well as those three mesenchymal markers, they were down-regulated by miR-124-3p overexpression (Figure 7C).

As a possible novel target of miR-124-3p, the function of ROCK1 in the epithelial mesenchymal transition was further assessed via RNAi approach. As we expected, decreased ROCK1 could up-regulate epithelial marker, β-catenin, and down-regulate three mesenchymal markers (Figure 7C). siROCK1 could simulate the effect of miR-124-3p over-expression and could reduce the expression of c-Met, MMP2, MMP9 as well (Figure 7C). The results indicated that overexpression of miR-124-3p represses the EMT phenotype of bladder cancer cells.

Furthermore, as we revealed that miR-124-3p could regulate the expression of c-Met, MMP2 and MMP9, we were interested in its underlying molecular mechanism. Previous studies showed that activation of RhoA/ROCK regulates NF-κB signaling pathway and RhoA/ROCK act upstream of p38 MAPK [22,23]. We performed western blot to explore whether miR-124-3p could affect these pathways. We found that both of miR-124-3p and siROCK1 could decrease the expression level of Phospho-p38 MAPK (p-p38) (Figure 7D). No significant change of NF-κB and Phospho-NF-κB was observed (data not show). In addition, we performed Immunohistochemical staining to assess the expression of MMP2 and MMP9 in human bladder cancer tissues, comparing with the paired non-tumor tissues. We confirmed that MMP2 and MMP9 were frequently up-regulated in bladder cancer tissues (Figure 7A, B). As we have revealed the down-regulation of ROCK1, MMP2 and MMP9 in bladder cancer tissues by IHC, we wanted to know whether knocking down miR-124-3p could result in the up-regulation of ROCK1, MMP2 and MMP9. Therefore, western blot was performed to investigate the expression of these genes after knocking down miR-124-3p by synthetic oligonucleotides. We found that ROCK1, MMP2 and MMP9 were up-regulated compared with the NC groups (Additional file 1: Figure S1). It may suggest that down-regulated miR-124-3p is one of factors which lead to the upregulation of these genes.

## Discussion

Previous studies showed that miR-124-3p is commonly down-regulated in several human cancers and transfection of it represses cell proliferation and motility of cancer cell lines [14-17]. Tumor-specific silencing of miR-124-3p was a relatively frequent molecular event in primary HCCs and miR-124-3p exert cell growth-inhibitory effect, resulting in cell cycle arrest at the G1–S checkpoint and apoptosis in cells [16]. The loss of miR-124-3p endows breast cancer cells higher capability in migration and invasion [17]. An earlier study showed that MicroRNA-124-3p is frequently methylated in all histological types of colorectal cancer and polyps [24]. Another study reported that miR-124-3p was frequently methylated in bladder cancer tissues, and the tumor tissues exhibited significantly higher methylation levels than their non-tumorous counterparts [19]. Thus, methylation may be an important mechanism which contributes to the down-regulation of miR-124-3p in bladder cancer tissues. However, the specific function of miR-124-3p in bladder cancer progression has not been fully understood.

In this study, we reported that the expression level of miR-124-3p was significantly lower in human bladder cancer cell lines and tissues, which is consistent with earlier study [19]. With the expansion of our bladder cancer tissues bank, further study at different levels would be designed to evaluate the clinical meaning of miR-124-3p in bladder cancer diagnosis, prognosis and treatment. In functional studies, the results of flow cytometry and colony-forming assay illustrated that transfection of miR-124-3p dramatically repress proliferation in bladder cancer cell lines, while no significant difference was observed between NC group and miR-124-3p-treated group in MTT assay. Therefore we think negative results occurred in viability study by MTT assay may be due to relatively short experiment time in comparison with clonogenicity assay. As described in materials and methods section, we assessed cell viability 24–96 h after transfection by MTT assay, while 14 days in clonogenicity assay. We tried to seed less amount of cells (approximately 500 cells/well) in each well. Nevertheless, basically 100% confluence in 96-well plates was reached within 5 days after RNAs transfection due to high speed proliferation of bladder cancer cells. If we further reduced the amount of cells, it might be a similar assay to assess clonogenicity of BCa cells. Therefore, despite the discordance of results of MTT assay with results of flow cytometry and clonogenicity assay, we suppose miR-124-3p may inhibit cell proliferation in bladder cancer cells. Reintroduction of miR-124-3p dramatically repressed the capability of migration and invasion in three human bladder cancer cell lines. These findings suggest that miR-124-3p plays a critical role in the invasive and metastatic potential of BCa and may be potential diagnostic and predictive biomarkers.

Subsequently, we demonstrated ROCK1 as direct target of miR-124-3p in human bladder cancer. Our findings show that miR-124-3p dramatically decreased their expression of ROCK1 in both mRNA level and protein level by bounding the complementary sites of its 3′-UTR. The co-transfection of pT-ROCK1 and miR-124-3p rescued the miR-124-3p induced repression in cell motility. ROCK1 is correlated with tumor migration and invasion and Rho/ROCK pathway participates in regulating cytoskeletal signalling [20,21,25,26]. Up-regulation of ROCK1 has been reported in bladder cancer and it has been verified to be associated with the progression of BCa [27]. This is consistent with our results that exogenetic overexpression of miR-124-3p suppress the migration and invasion of human bladder cancer cells. In addition, cell cycle, MTT assay and colony-forming assay presented no significant difference via RNAi approach of ROCK1, suggesting that there may be other target genes to regulate the proliferation of BCa cells in miR-124-3p downstream network. These observations provide the evidence that miR-124-3p exerts its function in cell motility and invasion via regulating the expression level of ROCK1.

It is generally accepted that invasion and metastasis are two of the most important hallmarks of malignant tumors. EMT is thought to be a key step in the progression of tumors toward invasion and metastasis. In the progress of EMT, epithelial cells gradually lose their epithelial adherence, tight junction, polarity, cell-cell contact and undergo remodeling of cytoskeleton, all of which promotes cell motility and invasion [28]. Rho/ROCK signaling has been reported to play a key role in the mediation of EMT [29]. In our study, we found that the mimics of miR-124 reverses EMT of T24 cell line, as shown by decreased expression of the mesenchymal markers like fibronectin, N-cadherin and vimentin while enhanced expression of the epithelial markers β-catenin. We tried to evaluate the expression of E-cadherin, another marker of epithelial cells. However it could not be detected by western blot. The hypermethylated E-cadherin promoter may prevent its expression [30]. Here, we confirmed that miR-124-3p reversed BCa cell EMT in vitro.

Next, we observed that the expression of c-Met, MMP2 and MMP9 were suppressed after reintroduction of miR-124-3p. c-Met, oncogene, is a well-characterized cell surface receptor tyrosine kinase and up-regulated in tumors, including human bladder cancer [31]. MMP2 and MMP9, downstream of c-Met, are two extracellular matrix-degrading enzymes [32,33]. They endow cancer cells high invasive and metastatic ability [34]. Silencing of ROCK1 by siRNA could simulate the effect of miR-124-3p over-expression, reversing EMT of T24 and repressing c-Met, MMP2, MMP9. Furthermore, our data suggested that miR-124-3p decreased p38 MAPK phosphorylation. In previous studies, p38 MAPK activation was demonstrated to regulate MMP2, MMP9 the activity of c-Met and can promote invasion of bladder cancer [35-37]. Therefore, miR-124-3p may regulate the expression of c-Met, MMP2, MMP9 through p38 pathway.

These results indicated that the loss of miR-124-3p gains the expression of ROCK1. Upregulated ROCK1 promotes BCa cells EMT which leads to migration and invasion through Rho/ROCK pathway. Although ROCK2 was reported as a target gene of miR-124 in HCC cells [38], we could not observe any expression difference of ROCK2 after miR-124-3p treatment by western blot in BCa cells (data not show). Thus, we considered that ROCK1 is more important in miR-124-3p-Rho/ROCK pathway in bladder cancer. It has been shown that Slug is another target gene of miR-124-3p, which participates in epithelial mesenchymal transition [17]. Therefore, the loss of miR-124-3p may also result in accumulation of Slug which promots EMT. On the other hand, ROCK1-induced high level of c-Met, MMP2 and MMP9, which enhances migration and invasion of bladder cancer cells. Furthermore, our preliminary data suggested SP1 maybe a new potential target gene of miR-124-3p. Bioinformatics prediction software Targetscan predicts that SP1 may have three binding sites of miR-124-3p. We found SP1 was down-regulated in BCa cells after miR-124-3p treatment by western blot (data not show). SP1 is a widely described gene involving in tumorigenesis, cancer metastasis and proliferation [39-42]. SP1 has a key role in initiation and propagation of EMT [43], and it could regulate the expression of Slug, c-Met, MMPs [44-46]. The results of previous studies of SP1 were consistent with our results after miR-124-3p transfection. It is necessary to take further experiments to illustrate miR-124-3p related downstream network in regulation of migration and invasion of BCa cells (Figure 7). As the important role of miR-124-3p in bladder cancer progression, we think further experiments in animal tumor models and clinical samples are necessary to determine the potential value of miR-124-3p in bladder cancer patients treatment in this era of translational medicine.

In conclusion, we confirmed that miR-124-3p works as a metastatic suppressor in BCa cells. The dysregulation of miR-124-3p gains the expression of ROCK1, which promotes the epithelial mesenchymal transfer and increases c-Met, MMP2, MMP2. These mechanisms endow the bladder cancer cells a higher capability in migration and invasion promoting tumor metastasis. We rationally speculate that miR-124-3p has the potential to be a useful clinical noninvasive diagnostics or predictive marker in human bladder cancer.

## Abbreviations

miRNA: MicroRNA; miR-124-3p: MicroRNA-124-3p; ROCK1: Rho-associated, coiled-coil containing protein kinase 1; qPCR: Quantitative RT-PCR assays; IHC: Immunohistochemical; NT: Non-tumor tissues; WT: Wild type; mut: Mutated; BCa: Bladder cancer; p-p38: Phospho-p38 MAPK; HCC: Hepatocellular carcinoma; EMT: Epithelial mesenchymal transfer.

## Competing interests

All authors declare that they have no competing interests.

## Authors’ contributions

XiaX, YL, HC, ZH performed and participated in analysis of laboratory experiments data. XiaX, YL, XZ, and LX participated in the design of experiments. XiaX, XinX, YM and JW acquired, preserved clinical samples. XZ, JL and LX provided administrative support and funded experiments. XiaX, YZ, SL, and LX drafted the manuscript. All authors have contributed and approved the final manuscript.

## Supplementary Material

Additional file 1: Figure S1 Western blot of ROCK1, MMP2 and MMP9 after miR-124-3p inhibitor treatment. GAPDH was used as loading control. Click here for file
