# Supplementary figures and images for: MicroRNA-124-3p inhibits cell migration and invasion in bladder cancer cells by targeting ROCK1
Source: J Transl Med. 2013 Nov 2;11:276. doi: 10.1186/1479-5876-11-276 (PMC4228407; doi:10.1186/1479-5876-11-276)

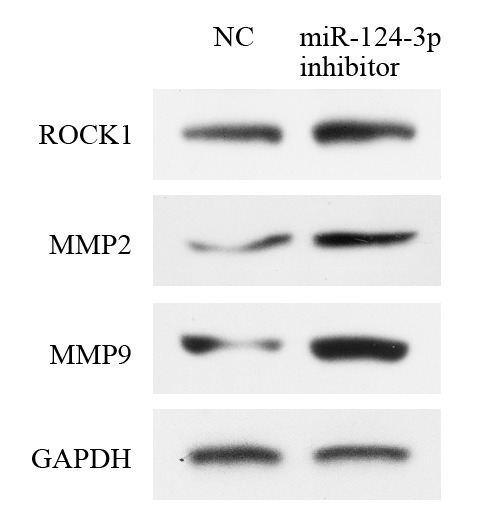

Supplement: Additional file 1: Figure S1 — Western blot of ROCK1, MMP2 and MMP9 after miR-124-3p inhibitor treatment. GAPDH was used as loading control. [file 1479-5876-11-276-S1.tiff]
